# Effects of Subject-Specific Robot-Guided Off-Axis Stepping Training in Individuals With Medial Knee Osteoarthritis

**DOI:** 10.1109/TNSRE.2026.3712232

**Published:** 2026

**Authors:** Raziyeh Baghi, Wei Yin, Song Joo Lee, Giovanni Oppizzi, Zongpan Li, Peter D. Bowman, R. Frank Henn, Li-Qun Zhang

**Affiliations:** Department of Physical Therapy and Rehabilitation Science, University of Maryland, Baltimore, Baltimore, MD 21201 USA. She is now with the Department of Physical Medicine and Rehabilitation, Johns Hopkins University, Baltimore, MD 21287 USA; Department of Physical Therapy and Rehabilitation Science, University of Maryland, Baltimore, Baltimore, MD 21201 USA. He is now with the School of Applied Engineering and Technology, New Jersey Institute of Technology, Newark, NJ 07102 USA; Bionics Research Center and the Division of Bio-Medical Science and Technology, Korea Institute of Science and Technology, Seoul 02792, South Korea; Department of Bioengineering, University of Maryland, College Park, MD 20742 USA; Department of Physical Therapy and Rehabilitation Science, University of Maryland, Baltimore, Baltimore, MD 21201 USA; Department of Physical Therapy and Rehabilitation Science, University of Maryland, Baltimore, Baltimore, MD 21201 USA; Department of Orthopaedics, University of Maryland, Baltimore, Baltimore, MD 21201 USA.; Department of Physical Therapy and Rehabilitation Science and the Department of Orthopaedics, University of Maryland, Baltimore, Baltimore, MD 21201 USA, and also with the Department of Bioengineering, University of Maryland, College Park, MD 20742 USA

**Keywords:** Osteoarthritis of knee, knee adduction moment, neuromuscular control, foot progression angle, biomechanics

## Abstract

Elevated peak knee adduction moment (pKAM) has been identified as a critical biomechanical factor driving medial knee osteoarthritis (KOA) initiation and progression, while impaired frontal plane neuromuscular control contributes to joint instability and accelerated disease advancement. Although gait modification strategies using real-time biofeedback have demonstrated pKAM reductions, most interventions apply the same modifications to all individuals and do not fully evaluate the combined effects of KAM reduction training and neuromuscular control training on joint loading, neuromuscular stability, and functional outcomes. A subject-specific robotic off-axis elliptical training system was developed to improve frontal plane neuromuscular control and reduce pKAM through foot progression angle (FPA) modification individually. Effects of the six-week subject-specific off-axis stepping training in reducing excessive knee loading and improving frontal-plane stability were demonstrated through 18 supervised training sessions in thirteen individuals with medial KOA. The training system perturbed the FPA slowly during continuous stepping without the subject noticing the perturbation to identify the optimal FPA for a targeted pKAM reduction. Furthermore, the optimized FPA was combined with real-time three-dimensional knee moment feedback and frontal plane footplate free-sliding challenges were used in the training. After the six-week training, participants demonstrated significant reductions in pKAM, sliding instability and maximum lateral sliding distance, while stepping speed increased significantly. Clinical outcomes showed improvements in KOOS Symptoms and Pain subscales. Functional performance enhanced in the 20-meter walk test, 30-second sit-to-stand test, overground gait velocity, and bilateral step length. Importantly, improvements in frontal plane stability metrics were maintained at six-week follow-up, suggesting lasting neuromuscular adaptations. The results indicate that robotic off-axis elliptical training with FPA optimization can potentially be used as a subject-specific therapeutic and research tool to train individuals with medial KOA for concurrent reductions in medial compartment overloading and improvements in frontal-plane neuromuscular control during functional weight-bearing stepping movements.

## I. Introduction

MEDIAL compartment knee osteoarthritis (KOA) is the most common form of KOA, characterized by gradual articular cartilage deterioration, leading to pain, stiffness, and functional limitations [1]. Abnormal knee joint loading, particularly elevated peak knee adduction moment (pKAM), has been identified as a key biomechanical factor contributing to medial KOA development and progression [2], [3]. Gait modification techniques, such as altering foot progression angle (FPA) using real-time biofeedback, have shown a 60% and 20% reduction in pKAM during walking [4], [5], [6], [7], [8]. However, most prior studies applied a uniform FPA across individuals, whereas recent work shows that individualized FPA strategies enable a higher proportion of participants to achieve pKAM reductions [9], [10]. For example, FPA modifications ranging from 25° toe-in to 30° toe-out have been explored [5], [8], [9], [11]. Testing numerous discrete FPA values is time-consuming and challenging, as individuals must continually adapt to each new foot-positioning strategy. Moreover, consciously modifying one’s FPA can lead to an unnatural gait pattern and altered knee mechanics, potentially limiting long-term adherence to the intervention.

A potential solution is to continuously vary the FPA within a toe-out to toe-in range during stepping *without* the subject’s awareness of these changes. This dynamic approach allows collection of many data points to estimate the linear relationship between FPA and pKAM for each individual, enabling determination of an optimal FPA that achieves a targeted pKAM reduction [12]. This method offers a more efficient and personalized way to identify beneficial gait modifications.

Neuromuscular control is crucial for maintaining joint stability and preventing abnormal loading [13]. In people with KOA, neuromuscular control—particularly in the frontal plane—is often impaired and may contribute to disease progression [14]. Knee joint instability is associated with greater pain and reduced physical activity. Bennell et al. demonstrated that a 12-week neuromuscular exercise program significantly improved knee function and reduced pain in KOA patients [15]. Additionally, a recent study reported that a single bout of frontal-plane gait perturbation improved muscle activation and slightly increased the knee flexion moment (KFM) [16]. Although the KFM increase had a small effect size after one session, a longer perturbation training regimen might produce more substantial improvements in knee loading.

Previous research has shown that gait modifications targeting one plane of motion can inadvertently alter loading in other planes [17], [18]. Specifically, Hunt et al. reported that toe-in gait patterns, while reducing KAM, can increase knee flexion moments [4]. Elevated knee flexion moment has been associated with increased patellofemoral joint stress and anterior knee pain [19], [20], while excessive pKIRM may contribute to tibiofemoral cartilage shear stress and ligamentous strain [21]. Therefore, assessing these secondary moments is critical for evaluating the safety profile of the intervention and ensuring that pKAM reduction does not occur at the expense of creating new biomechanical risk factors for joint damage or pain [22].

Determining an appropriate target for pKAM reduction requires consideration of how external knee moments relate to overall joint loading. Manal et al. demonstrated that while pKAM is the primary predictor of medial knee contact force (mKCF) during walking (R^2^ = 0.63), pKFM also contributes significantly, explaining an additional 13% of variance in the regression model [23]. Importantly, other evidence revealed that for a 10% reduction in pKAM to effectively decrease mKCF, any concurrent increase in pKFM must remain below approximately 23% [24]. In general, KOA may involve transverse plane as well as frontal plane overloading. Furthermore, knee injuries like ACL injury may involve excessive tibial rotation overloading, and knee injuries may lead to traumatic KOA. Therefore, 3-D knee moment overloading should be investigated.

While various gait modifications and exercise interventions have been investigated for their effects on knee loading and clinical outcomes in medial KOA, the efficacy of a comprehensive program specifically targeting KAM reduction *and* frontal-plane stability improvement remains unclear. A customized robotic elliptical stepping system with perturbation capabilities and real-time knee moment estimation has been developed to facilitate such training [12], [25], [26]. This system can slowly rotate the footplates through different angles to identify an individual’s optimal FPA for reducing KAM, providing a novel approach to personalized gait rehabilitation with precise control and measurement capabilities. The purpose of this study was to evaluate the effects of a six-week robot-guided off-axis stepping training program using an individualized FPA (selected to achieve at least a 10% reduction in pKAM) on knee joint moments, frontal-plane stability, and functional outcomes in individuals with medial KOA. We hypothesized that pKAM and knee adduction moment impulse (ImpKAM) would decrease after training, while peak knee flexion moment (pKFM) and peak knee internal rotation moment (pKIRM) would not increase post-training.

## II. Material and Methods

### A. Study Participants

Thirteen individuals with symptomatic medial KOA were recruited for this study. Inclusion criteria included individuals between 50 and 80 years old with physician-diagnosed KOA, medial compartment knee pain at least once per week in the past six weeks, and radiographic evidence of medial KOA (Kellgren-Lawrence grade ≥ 1). Due to evaluation system setup, only participants with right-side KOA (or bilateral KOA with the right side more affected) were included.

Exclusion criteria included significant hip or ankle injury affecting gait; recent (≤ 6 months) intra-articular knee injections or corticosteroid use; history of knee or hip replacement; neurological disorders; severe cardiovascular disease; or uncontrolled hypertension. The study was approved by the Institutional Review Board of the University of Maryland Baltimore, with the protocol number HP-00082206, and all participants provided informed consent.

### B. Real-Time Knee Moment Determination and Off-Axis Sliding Stability Assessment System

**Fig. 1.**
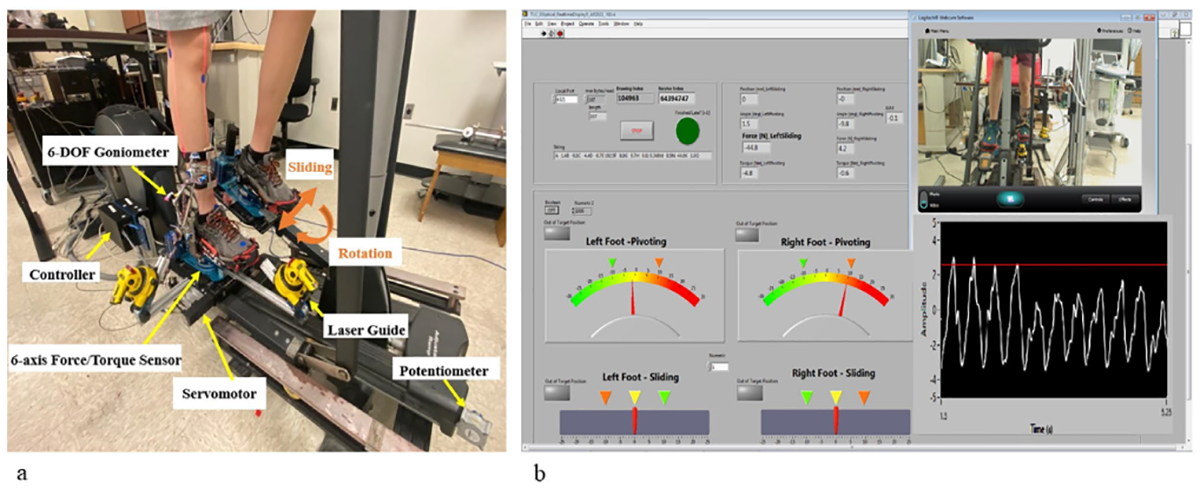
Robotic off-axis elliptical stepping system. (a) Components of the off-axis stepping system and the anatomical ankle zero-angle calibration. Footplate motion is motor-controlled in the transverse and frontal planes. Reaction forces and moments are measured by the 6-axis force/torque sensors, and ankle kinematics are recorded via the 6-DOF goniometer. To calibrate ankle “zero” angles, the participant adjusts lower-leg position using a laser alignment tool: in the frontal plane, the center of the JR3 force sensor is aligned with the second metatarsal head, the midpoint between the malleoli, and the tibial tuberosity. (b) Proper lower-leg alignment during elliptical stepping is demonstrated to participants via a video feed. Real-time visual feedback of footplate position in the frontal and transverse planes during stepping (in both spring-resisted and free-sliding modes) was provided to facilitate alignment.

We utilized a custom-built robotic off-axis elliptical stepping system capable of controlled motion in the sagittal, frontal, and transverse planes to train and evaluate frontal-plane stability (Fig. 1a). Detailed system specifications are available in [27] and [28].

For real-time knee moment measurement, the system integrates a 6-DOF goniometer to record 3D ankle angles and six-axis force/torque sensors (JR3, Woodland, CA) mounted under each footplate (Fig. 1a). Kinematic and kinetic data were sampled at 100 Hz and fed into a custom 3D inverse dynamics program for real-time estimation of knee moments [25], [26]. Further details on moment computation are available in [25], [26], and [29].

### C. Outcome Measures and Testing Procedures

All outcome measures described below were assessed at three time points: before training (baseline), immediately after completing the six-week training program (post-training), and six weeks after training completion (follow-up).

#### 1) Target FPA Determination and Knee Moment Assessment:

**Fig. 2.**
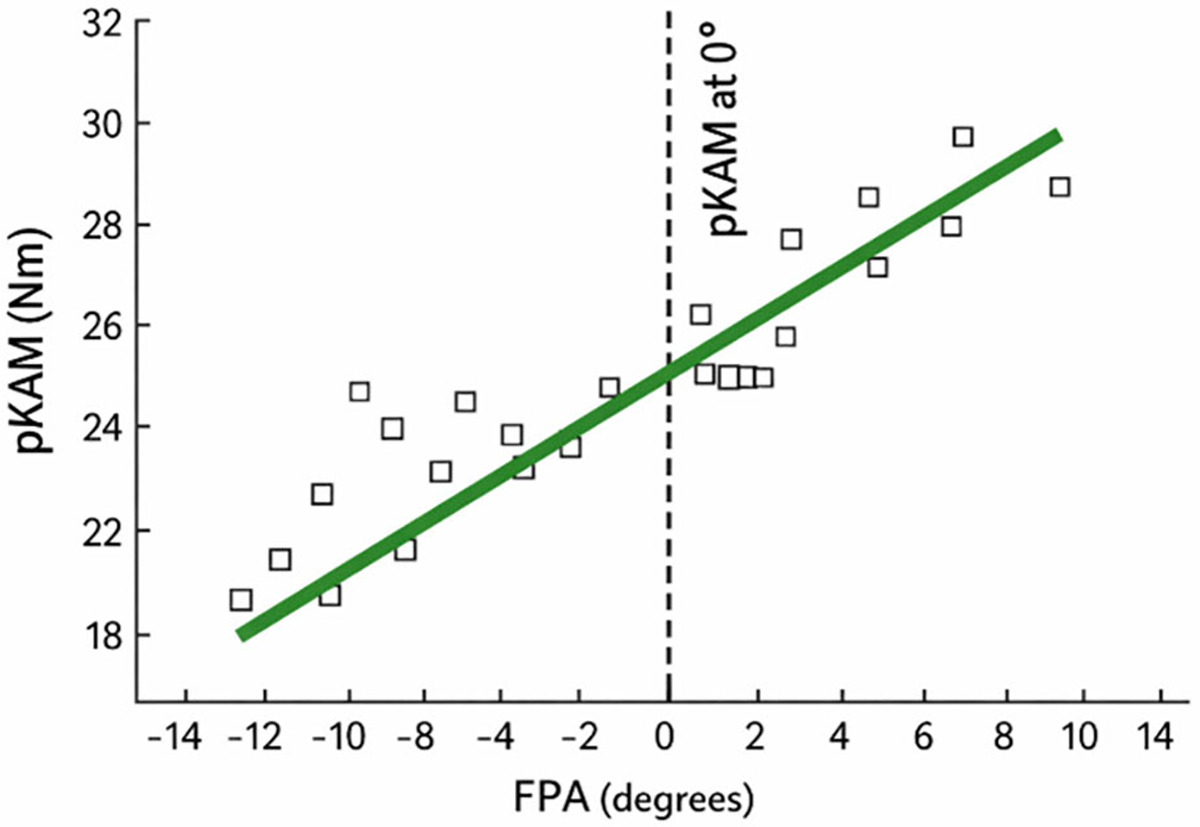
Typical relationship between pKAM and the FPA for a representative study participant. Using this relationship, the optimal FPA was detected for the training sessions. pKAM: peak knee adduction moment, FPA: foot progression angle.

**Fig. 3.**
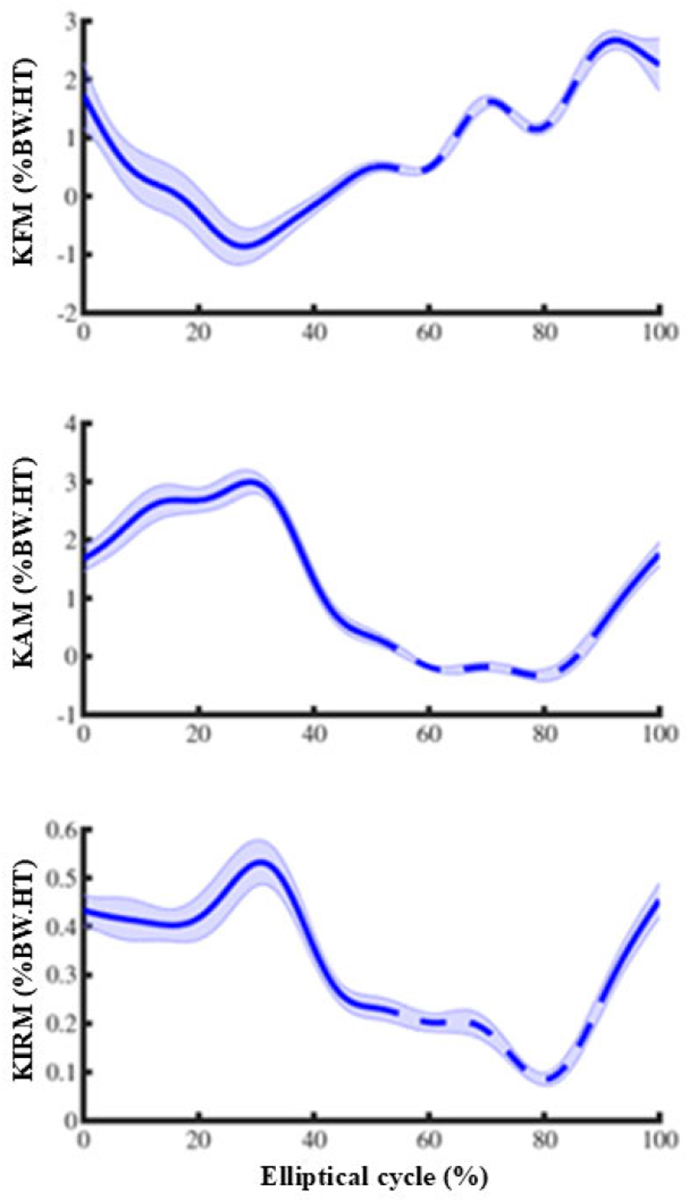
Typical knee moment components during elliptical stepping in a representative study participant, with the footplates locked at 0° FPA. KF: knee flexion moment, KAM: knee adduction moment, KIRM: knee internal rotation moment, BW: body weight, H: height.

Baseline testing included a procedure to determine each participant’s target FPA—the foot rotation angle predicted to reduce pKAM by ~10%. Participants stepped continuously on the device at a self-selected pace while the footplates were slowly and smoothly rotated from 10° toe-out to 10° toe-in (~0.5°/s). This gradual continuous change in footplate orientation enabled participants to maintain a consistent stepping pattern and rhythm without perceiving distinct footplate perturbations. As the footplate moved between 10° toe-out and 10° toe-in, pKAM was computed for each stepping cycle. We then examined the linear relationship between FPA and pKAM by calculating the slope and intercept of the pKAM–FPA curve (Fig 2). The slope represented the rate of change of pKAM per degree of FPA, and the intercept corresponded to the estimated pKAM at 0° FPA. Using these parameters, we identified the subject-specific FPA predicted to achieve a 10% reduction in pKAM (described in Section D). At all three evaluation time points (baseline, post-training, and follow-up), knee joint moments were measured during stepping with neutral FPA (i.e., 0°, standard foot position) (Fig 3). Outcome measures included pKAM, pKFM, pKIRM, and ImpKAM.

#### 2) Frontal Plane Stability Assessment:

At all three time points, frontal-plane stability of the right leg was assessed as the ability to control the mediolateral position of the right footplate under a low-resistance “free sliding” mode (i.e. backdrivable). In free-sliding mode, the footplate can move freely side-to-side with minimal resistance, challenging participants to actively control footplate position during stepping. Participants were instructed to maintain the footplate’s initial neutral mediolateral position while stepping continuously for one minute. A visual target indicating the neutral position was displayed on a monitor to provide real-time feedback and guide participants in keeping the footplate centered.

Frontal-plane stability performance was quantified using three metrics:

- **Sliding instability:** the standard deviation of the footplate’s mediolateral displacement (with a smaller value indicating better stability/control).
- **Maximum lateral displacement and maximum medial displacement:** the farthest distances the footplate moved laterally and medially from neutral (smaller values also indicating better control).

Overall, lower values in these measures correspond to better neuromuscular control in the frontal plane.

#### 3) Clinical and Functional Outcome Measures:

Clinical outcomes were assessed via the Knee injury and Osteoarthritis Outcome Score (KOOS) questionnaire, which evaluates knee status across five subscales: Pain, Symptoms, Activities of Daily Living (ADL), Sport and Recreation Function, and knee-related Quality of Life (QOL). Each KOOS subscale is scored 0–100 (0 = extreme knee problems, 100 = no problems).

Functional performance was evaluated with four standard tests: the 30-second sit-to-stand test (STS), the Timed Up and Go (TUG), the 20-meter walk test (20MWT), and the Four-Square Step Test (FSST). In addition, spatiotemporal gait parameters were recorded during overground walking using a GAITRite^®^ electronic walkway (5.4 m length). Gait measures included walking velocity, cadence, step lengths (right and left), step-length symmetry, single-limb support phase (right and left), and single-limb support symmetry.

### D. Training Protocol

Participants underwent 18 supervised training sessions (3 sessions per week for 6 weeks) using the off-axis elliptical trainer. To ensure safety, a ceiling-mounted harness was worn to prevent falls (without supporting body weight), and exercise intensity was monitored via Borg Rating of Perceived Exertion scale, heart rate, and blood oxygen level via finger pulse oximeter to minimize cardiovascular risk. Training was temporarily paused if any of the following occurred: RPE >15 (“hard”), heart rate >85% of age-predicted maximum (220 - age), SpO2 < 90%, or participant-reported discomfort. Blood pressure was measured before and after each session to ensure participants remained within safe limits (systolic <180 mmHg, diastolic <110 mmHg).

**Fig. 4.**
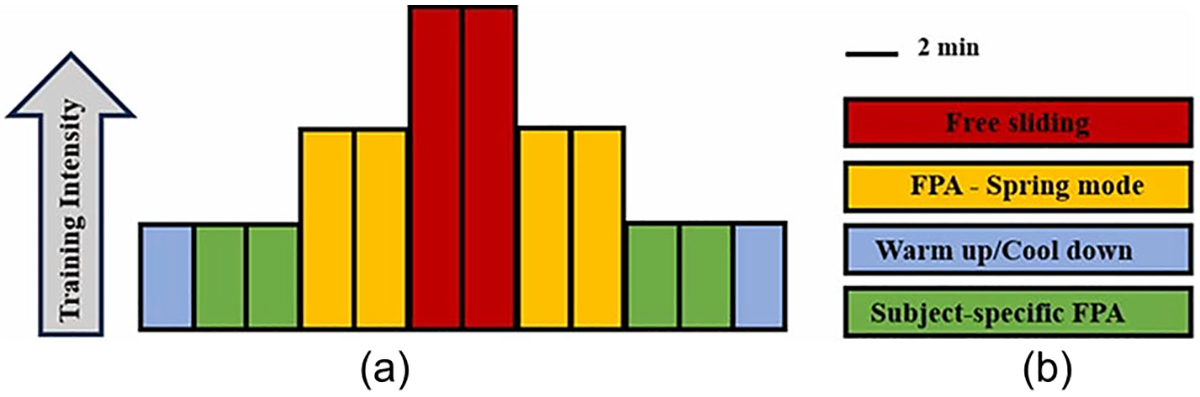
Diagram of a typical training session. The session started with a warm-up phase of stepping with the footplates in the neutral position (0° FPA). This was followed by a phase of stepping with the footplates locked at the subject-specific target FPA. In the third phase, the footplate was set to spring mode, providing a resistive lateral force that the participant had to counter to maintain the target FPA. The fourth phase involved stepping in free-sliding mode (no resistance in the frontal plane). The session concluded with a cool-down phase stepping again at the neutral footplate position (0° FPA).

Each training session followed a standardized protocol consisting of four sequential phases (Fig. 2), with total active stepping time of 30 minutes (excluding rest breaks). A typical training session consisted of the warm-up phase, subject-specific FPA, FPA-Spring phase, and free-sliding phase is shown in Fig. 4.

#### 1) Warm-Up Phase (2 Minutes):

Participants completed stepping with both the footplate rotation and sliding mechanisms locked in neutral position (0° FPA, centered mediolateral position) at a self-selected comfortable pace.

#### 2) Fixed Target FPA Phase (8 Minutes):

The footplates were locked at each participant’s individualized target FPA (determined during baseline testing as described in Section C.1). Participants stepped continuously at their self-selected pace while receiving real-time visual feedback of their estimated pKAM displayed on a monitor. This phase familiarized participants with the modified foot position and allowed them to observe the associated pKAM reduction. Participants were given a 1–2 minute rest break after this phase if requested.

#### 3) FPA-Spring Resistance Phase (8 Minutes):

The right footplate was switched to spring mode, which applied lateral resistance tending to push the footplate back toward neutral (0° FPA). Participants were instructed to actively maintain their foot position aligned with a yellow target line indicating their individualized FPA (see Fig. 1b) while stepping. The target FPA was adjusted for each of the individuals to reduce the pKAM, and it ranged from −10° to 10° across the participants and remained about fixed across the training sessions. Concurrent real-time visual feedback of estimated pKAM was displayed on the same screen, enabling participants to associate the maintained foot position with reduced knee loading. This phase progressed from continuous 2-minute bouts with 1-minute rest intervals in weeks 1–3, to 3–5minute bouts with 1-minute rest in weeks 4–6, contingent on participant tolerance and safety monitoring criteria.

#### 4) Frontal-Plane Stability Training Phase (4 Minutes):

The right footplate was switched to free-sliding mode (backdrivable with minimal resistance), allowing mediolateral movement. Participants received continuous audiovisual feedback consisting of: (a) a visual target line on the monitor indicating the neutral mediolateral position, (b) real-time display of footplate position and lower limb alignment captured by video camera, and (c) verbal encouragement from the investigator to maintain centered position. Participants were instructed to actively control mediolateral footplate position to keep it centered while stepping continuously. This phase also progressed from 2-minute bouts with 1-minute rest intervals in weeks 1–3, to 3–5minute bouts in weeks 4–6, based on participant tolerance.

#### 5) Cool-Down Phase (2 Minutes):

Similar to the warm-up phase, participants completed stepping with both the footplate rotation and sliding mechanisms locked in neutral position at a self-selected comfortable pace.

Total session duration, including setup, monitoring, and rest periods, was approximately 45–50 minutes.

### E. Statistical Analysis

Normal distribution was assessed using Shapiro-Wilk test. Repeated measure ANOVA were used to evaluate the impact of the 6-week training on key outcome measures by comparing the variables between the 3 evaluation timepoints: stepping speed, sliding instability, maximum medial and lateral sliding displacements, pKAM, pKFM, pKIRM, ImpKAM during stepping, KOOS subscale scores, TUG, 20MWT, FSST, and spatiotemporal gait parameters. For the only ordinal variable in the study, STS, Friedman test was utilized. A Bonferroni correction was applied for multiple comparisons within each category of outcomes, and the significance level was set at *p* < 0.05.

## III. Results

Thirteen participants with symptomatic medial knee osteoarthritis (KOA) (10 females, 3 males; age: 64.76 ± 9.78 years; mass: 87.79 ± 15.70 kg; height: 1.67 0.07 m; body mass index: 31.85 ± 6.91 kg/m^2^; Kellgren-Lawrence grade: 2.29 ± 1.09, KOA side: bilateral: 7, right side: 5, left side: 1) completed 18 training sessions over 6 weeks (3 sessions/week). All participants underwent baseline and post-training evaluations, with eight completing an additional follow-up evaluation at 6 weeks post-training.

### A. Frontal Plane Stability Improvements

**Fig. 5.**
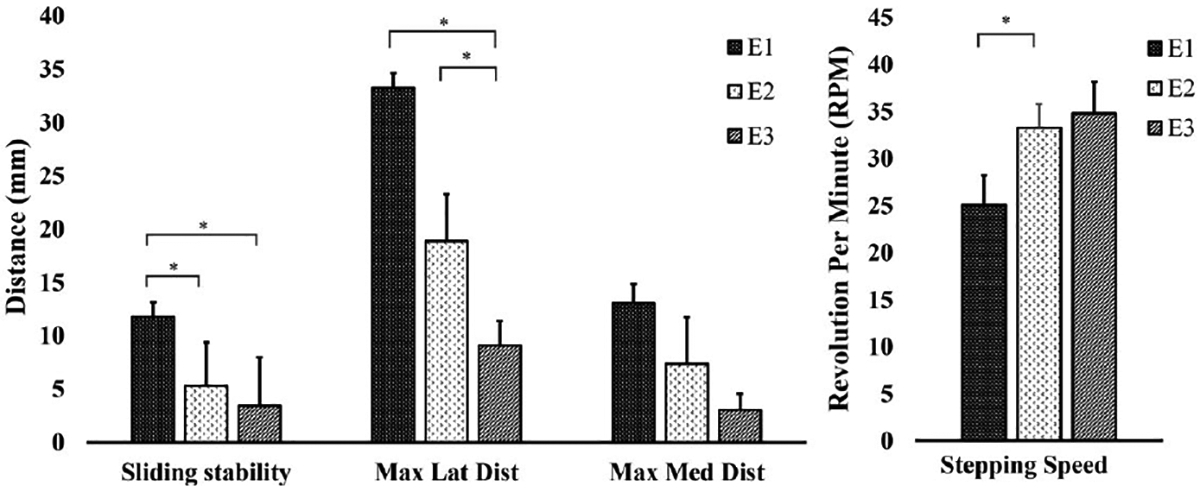
Frontal plane stability improvements with training. (a) Sliding instability (Instability), maximum lateral sliding distance (Lat Slide Dist), and maximum medial sliding distance (Med Slide Dist) during free-sliding stepping at baseline, post-training, and 6-week follow-up. (b) Stepping speed (in revolutions per minute, RPM) at baseline, post-training, and 6-week follow-up. Error bars indicate standard error of the mean. E1: Baseline evaluation, E2: Post-training evaluation, E3: Follow-up evaluation. Statistically significant difference compared to baseline (*p* < 0.05).

The 6-week off-axis stepping training led to significant improvements in frontal-plane stability. Sliding instability (the standard deviation of mediolateral footplate motion) decreased from 11.76 ± 5.05 mm at baseline to 5.29 ± 5.04 mm post-training (*p* = 0.005). This improvement was largely retained at follow-up (3.40 ± 4.99 mm; baseline vs. follow-up *p* = 0.044) (Fig. 5a).

Maximum lateral sliding distance (the largest excursion of the footplate to the outside) was reduced from 33.24 ± 14.71 mm at baseline to 18.88 ± 15.88 mm post-training (*p* = 0.043, *d* = −0.88), and further to 9.10 ± 12.37 mm at follow-up (baseline vs. follow-up *p* = 0.049, *d* = −1.19) (Fig. 5a). In contrast, maximum medial sliding distance did not change significantly (baseline 13.09 ± 16.48 mm, post-training 7.34 ± 8.28 mm, follow-up 3.05 ± 4.22 mm; *p* = 0.801) (Fig. 5a). Stepping speed (elliptical stride rate) increased from 25.11 ± 11.15 RPM at baseline to 33.25 ± 9.10 RPM post-training (*p* = 0.029) and remained slightly elevated at follow-up (34.79 ± 8.90 RPM; not significantly different from baseline, *p* = 0.178) (Fig. 5b).

### B. Improvements in Knee Moment

**Fig. 6.**
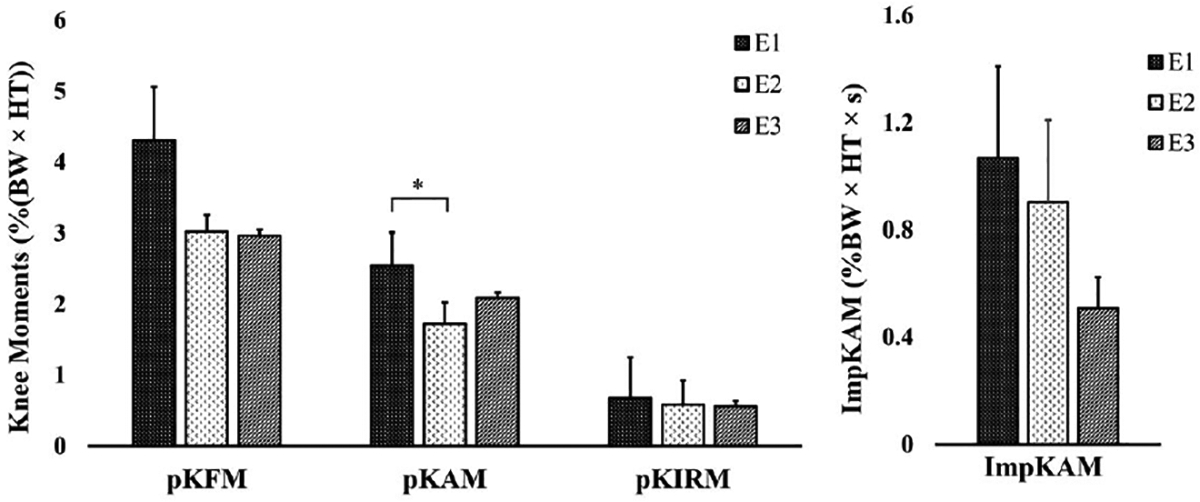
Peak knee flexion moment (pKFM), peak knee adduction moment (pKAM), peak knee internal rotation moment (pKIRM), and knee adduction moment impulse (ImpKAM) at baseline, post-training, and 6-week follow-up. E1: Baseline evaluation, E2: Post-training evaluation, E3: Follow-up evaluation, BW: body weight, HT: height, s: seconds. Error bars represent standard error of the mean. Significant difference from baseline.

**Fig. 7.**
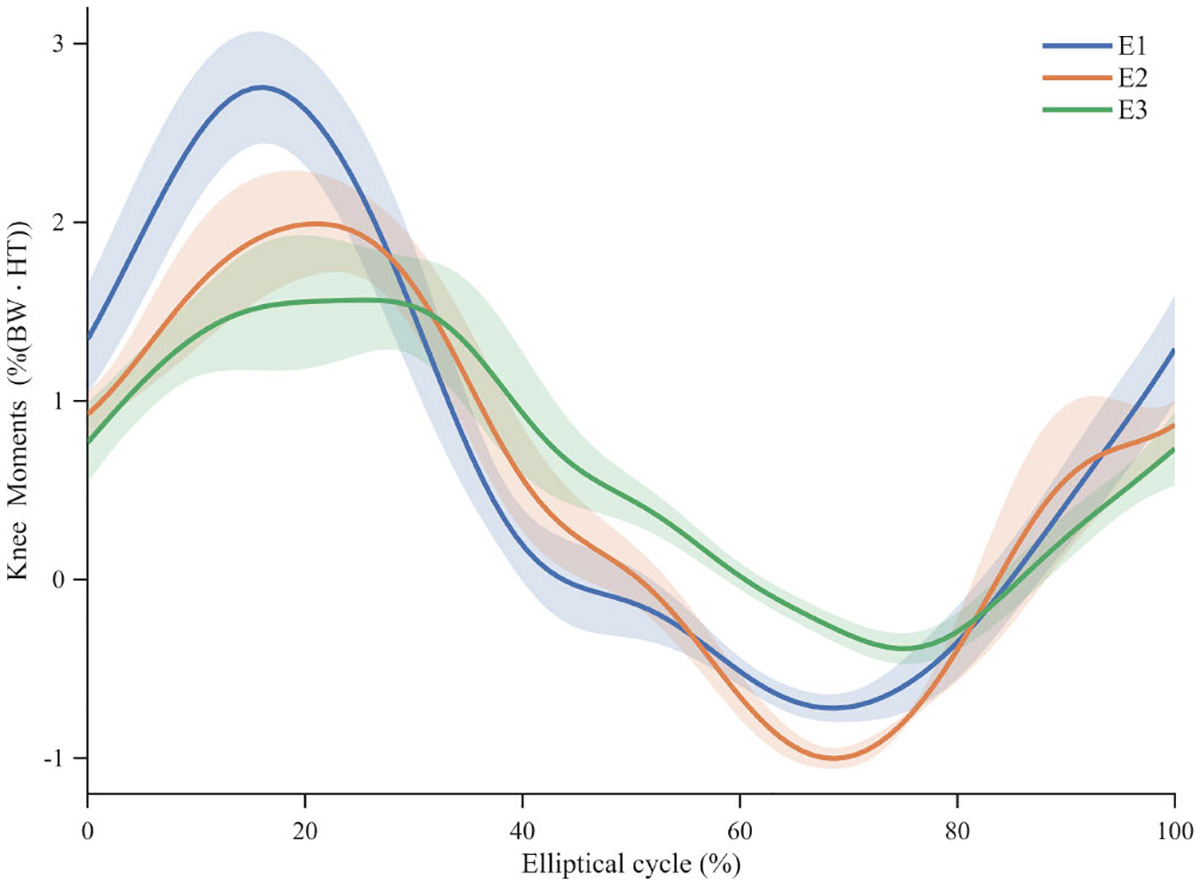
Changes in the KAM during stepping between different evaluation timepoints in a representative subject. KAM: knee adduction moment, E1: Baseline evaluation, E2: Post-training evaluation, E3: Follow-up evaluation, BW: body weight, HT: height.

The off-axis training produced a substantial reduction in the knee adduction moment. Peak KAM (first peak in the stance phase during stepping) decreased by 33.03 ± 25.47% on average, from 2.54 ± 0.71 %BW·HT at baseline to 1.72 ± 0.91 %BW·HT post-training (*p* = 0.011). At the 6-week follow-up, mean pKAM was 2.09 ± 0.90 %BW·HT, which was still lower than baseline but not significantly different from it (*p* > 0.99) (Fig. 6 & Fig 7). Knee adduction moment impulse (ImpKAM) showed no significant change post-training (baseline 1.07 ± 1.03 %BW·HT·s vs. post 0.90 ± 0.92 %BW·HT·s) or at follow-up (0.51 ± 0.33 %BW·HT·s) (*p* = 0.51). Importantly, the training did *not* cause any undesired increases in the other knee joint moments. Peak KFM actually decreased (baseline 4.31 ± 2.26 %BW·HT, post 3.02 ± 1.40 %BW·HT), though this reduction did not reach significance (*p* = 0.146). Peak KIRM changed only slightly (baseline 0.67 ± 0.26 %BW·HT, post 0.58 ± 0.22 %BW·HT; *p* = 0.125). None of the changes in pKFM or pKIRM were statistically significant, and at follow-up both remained similar or lower than baseline values (Fig. 6).

### C. Clinical and Functional Improvements

Participants reported improved knee symptoms after training. The KOOS Symptoms subscale increased from 63.89 ± 15.09 at baseline to 74.21 ± 13.10 post-training (*p* = 0.003), indicating reduced symptoms (higher scores reflect better outcomes). There were no significant improvements in the KOOS Pain, KOOS ADL, Sport/Rec, or QOL subscales (all *p* > 0.05). Table I provides the KOOS results at all time points

Functional performance tests showed select improvement post-training. The STS repetitions increased by 18.29 ± 13.24% (9.00 ± 2.83 to 10.33 ± 3.01, *p* = 0.004). In contrast, TUG time showed a non-significant trend towards improvement (11.12 ± 1.27 s to 9.82 ± 1.60 s, *p* = 0.249). Similarly, the 20MWT time showed a non-significant trend toward improvement (baseline 19.36 ± 2.24 s vs. post 17.33 ± 3.15 s, *p* = 0.071). FSST performance did not change significantly (9.50 ± 1.12 s vs. 8.50 ± 0.88 s, *p* = 0.192). These results are summarized in Table II.

### D. Spatiotemporal Characteristics in Overground Walking

Changes in overground gait parameters were also observed. Because only 4 participants completed the follow-up gait assessment, statistical comparisons were made primarily between baseline and post-training (with follow-up values provided descriptively in Table III). Walking velocity increased from 1.09 ± 0.26 m/s at baseline to 1.18 ± 0.25 m/s post-training (*p* = 0.060). Right step length increased from 62.40 ± 9.65 cm to 64.97 ± 9.27 cm (*p* = 0.052), while left step length also showed significant changes (62.84 ± 9.38 cm vs. 65.52 ± 8.42 cm, *p* = 0.314). Step-length symmetry was essentially unchanged (about 0.99 at both time points). The duration of single-limb support for right and left legs, and the symmetry of single support, also remained unchanged post-training (all *p* > 0.20). Table III details the spatiotemporal gait outcomes.

## IV. Discussion

This study evaluated a novel six-week, subject-specific off-axis stepping training program and found significant improvements in several biomechanical and clinical measures in individuals with medial KOA. The key findings included reduced knee adduction moment and frontal-plane instability, along with improvements in certain self-reported symptoms, functional test performance, and gait parameters. These results suggest that the innovative training approach used here may offer a promising new option for managing medial KOA.

### A. Reduction in pKAM

A primary finding was a significant ~33% reduction in pKAM after training, which is clinicallŷ meaningful given that pKAM is strongly linked to the progression of medial KOA [15], [30]. The reduction in pKAM likely resulted from using an individualized FPA during training, chosen to achieve at least a 10% pKAM decrease. This aligns with previous gait retraining studies showing that toe-out and toe-in modifications can reduce the knee adduction moment (second and first peak, respectively) and improve pain and function. Our robotic elliptical system enabled rapid identification of each subject’s optimal FPA via the continuous FPA perturbation method, avoiding the need for laborious trial-and-error testing of discrete angles. By automating FPA adjustments and providing real-time feedback, the system reduced the burden on both participants and clinicians in finding an optimal gait modification. Future research could extend this approach by also incorporating step-width modifications in combination with FPA changes to further offload the medial compartment.

### B. Changes in pKFM

Although pKFM did not significantly change post-training, we observed a trend towards reduction. Reductions in both pKAM and pKFM are desirable, as lowering these moments can lessen medial knee contact forces and potentially slow KOA progression [23], [31]. The trend toward reduced pKFM may indicate a smoother, more efficient stepping pattern resulting from training. The off-axis elliptical trainer’s design, with multi-planar movement control, might promote a more balanced force distribution across the knee, thereby avoiding excessive sagittal-plane loading. By encouraging a more natural, evenly distributed loading pattern, the device could mitigate undue stress on anterior knee structures during exercise [32], [33]. Prior studies of elliptical exercise have noted that it can produce lower joint loads compared to treadmill walking, which might partly explain the non-significant reduction in pKFM we observed. In any case, it is important that the training did not increase pKFM or pKIRM, since an increase in these could counteract the benefits of reducing pKAM.

### C. Changes in pKIRM

Our findings did not show a significant change in pKIRM following training, with values remaining similar or lower than baseline at follow-up. This finding is clinically important as it demonstrates that the subject-specific FPA optimization did not inadvertently increase transverse plane loading, a key safety consideration given that elevated pKIRM has been associated with increased tibiofemoral cartilage shear stress and ligamentous strain [21]. Previous research has shown that gait modifications targeting one plane can produce unintended alterations in other planes [17], [18], yet our intervention achieved significant pKAM reduction without compromising transverse or sagittal plane mechanics. The absence of increased pKIRM, combined with the non-significant reduction in pKFM, confirms that the training met our hypothesis that secondary moments would not increase post-training, supporting the biomechanical safety and efficacy of this multi-planar approach to medial KOA management.

### D. Frontal Plane Stability

Frontal-plane stability improved substantially following the training, as evidenced by reduced sliding instability, decreased maximum lateral sliding distance, and increased stepping speed, with many of these gains maintained at the 6-week follow-up. These results are consistent with previous studies using off-axis stability training in various populations (healthy individuals, patellofemoral pain syndrome, knee injury patients, KOA, and cerebral palsy), which have demonstrated enhanced pivoting/sliding stability, improved proprioception, and better functional outcomes [28], [34], [35], [36], [37], [38], [39]. The improvements in our study likely stem from the challenging nature of the free-sliding perturbation, which required participants to actively engage neuromuscular control to maintain stability, combined with the real-time visual feedback that guided proper movement and alignment. The continuous feedback provided participants with immediate knowledge of results, facilitating the adoption of strategies to keep the footplate stable. This kind of augmented feedback is known to accelerate motor learning and was an integral part of our training design.

### E. Clinical and Functional Outcomes

Participants experienced a significant short-term reduction in knee symptoms as reflected in the KOOS Symptoms and KOOS Pain subscales, corroborating prior evidence that targeted exercise can alleviate pain and symptoms in knee OA [40], [41], [42]. Our KOOS improvements are comparable to or larger than those reported in similar interventions. For instance, Lee et al. observed only slight KOOS gains in a pilot off-axis training with a small KOA sample [35]. In contrast, our study (with a larger sample) showed an ~11.16-point increase in KOOS Symptoms post-training, which exceeds the commonly cited minimal clinically important difference (MCID) of about 8–11 points for KOOS in knee disorders [43], [44]. The KOOS Pain subscale did not show a significant improvement. However, since we used repeated measure ANOVA, data for 8 subjects was included in analysis. When we used paired t-test for comparison of pre- and post-training evaluation timepoints of 13 study participants, a significant improvement in pain was observed (~8.64 points, *p* = 0.028) suggested the efficacy of our training in improving knee OA symptoms. By 6-week follow-up, the KOOS gains had partially regressed (Symptoms subscale + 4.16 points above baseline, which is below MCID). It should be noted that fewer participants completed the follow-up, and one individual had a pain flare due to fibromyalgia, which may have dampened the group’s average follow-up scores.

Although only STS showed significant improvement, we still observed changes in functional tests like the 20MWT and, indicating enhanced mobility and lower-limb function. The 10% faster 20m walk time and 17% higher sit-to-stand count suggest improved muscular strength/endurance and confidence in daily tasks. One of the explanations for the absence of significant improvement in all functional measures can be attributed to the high baseline function among participants. Participants with lower baseline function might experience even greater benefits, as our group was relatively high functioning to begin with. These functional gains likely result from the combined effects of reduced pathological KAM loading (making movement less painful), improved frontal-plane stability (reducing fear of instability or falls), and the general conditioning achieved through six weeks of elliptical exercise.

### F. Spatiotemporal Gait Parameters

The training program led to significant improvements in certain spatiotemporal gait parameters during overground walking. Walking velocity and step length increased post-training, consistent with previous reports that exercise interventions can improve gait in KOA [15], [45]. For example, Al-Khlaifat et al. found increases in walking speed and step length after a 6-week combined strengthening and gait retraining program in medial KOA patients [46]. To our knowledge, our study is the first KAM-focused retraining study to assess both patient-reported outcomes and objective gait measures in KOA. The gait enhancements we observed may be attributed to the reduction in medial knee loading (KAM) and better frontal-plane stability achieved by training, which together could allow a more confident and efficient walking pattern. In essence, by simultaneously addressing knee joint loading and stability, the program may have removed some constraints that previously limited the participants’ walking speed and stride length.

The present intervention applied individualized FPA modification during robotic elliptical stepping rather than during overground walking. A key question is whether the trained adjustment can transfer to daily walking in real-world environments. Although elliptical stepping differs from overground gait in terms of continuous foot contact and constrained trajectory, several features of the training paradigm may facilitate transfer. First, the intervention provided repeated, task-specific exposure to the individualized FPA under full weight-bearing conditions, coupled with real-time visual feedback of knee adduction moment. This combination likely promoted motor learning through reinforcement of a biomechanically advantageous lower-limb alignment rather than reliance on conscious foot placement strategies alone.

Second, the training emphasized frontal-plane neuromuscular control through free-sliding perturbations, which may generalize across locomotor tasks by improving sensorimotor coordination, proprioception, and dynamic limb control. Improvements in these underlying neuromuscular capacities could support more stable and efficient limb positioning during overground walking, even in the absence of explicit FPA feedback.

Importantly, although participants were not instructed to consciously modify their FPA during overground walking, we observed significant post-training improvements in walking velocity and step length, suggesting functional transfer beyond the training context. While knee moments were not directly measured during overground gait in this study, these spatiotemporal improvements are consistent with prior work showing that reduced medial knee loading and improved frontal-plane control can facilitate a more confident and efficient walking pattern.

Nevertheless, it remains unclear to what extent participants spontaneously adopted their individualized FPA during daily walking, or whether the observed gait improvements reflect indirect benefits of improved strength, stability, and reduced pain rather than direct retention of the trained foot rotation.

### G. Limitations

Several limitations should be considered when interpreting our findings. First, the sample size was small (N = 13 completing training, with only 8 completing follow-up evaluation), which limits the statistical power and the generalizability of results to broader KOA populations with greater demographic diversity, disease severity, and comorbidity profiles. The high attrition rate also introduced potential bias if participants who completed follow-up differed systematically from those who did not. Although significant differences were observed for the primary outcomes (pKAM and sliding instability), these estimates should still be interpreted with caution given the small sample size and associated imprecision. Nevertheless, the magnitude and consistency of the observed biomechanical changes suggest that the intervention could produce clinically meaningful adaptations, supporting its feasibility and potential efficacy. Future studies with more diverse cohorts and improved retention would help corroborate these preliminary findings.

**Second**, our study did not include a control group. Without a control group, improvements in pKAM, frontal plane stability, KOOS subscales, and functional performance may partially reflect non-specific factors such as increased attention and supervision, expectation/placebo effects, familiarization with the device and testing procedures (learning effects), or natural fluctuations in KOA symptoms over time. In addition, repeated exposure to the stepping assessments and real-time feedback could contribute to performance gains independent of the specific subject-specific FPA optimization and free-sliding stability training components. Therefore, the observed changes should be interpreted as promising preliminary evidence rather than definitive proof of efficacy. Future studies should include an appropriate control/comparison group (e.g., usual care, conventional elliptical exercise, or non-personalized FPA training) and ideally use a randomized controlled design to isolate the intervention-specific effects and quantify added benefit beyond general exercise and repeated testing.

**Third**, another limitation is the moderate length of the 6-week follow-up period. While the retention of improvements in frontal-plane stability metrics at follow-up is encouraging and suggests short-term neuromuscular adaptation, the durability of the observed biomechanical and clinical benefits over longer time scales remains unknown. Neuromuscular control adaptations, particularly those involving frontal plane stability and sensorimotor integration, may persist longer than changes in pain or self-reported function, which are more susceptible to daily activity levels, symptom fluctuation, and disease progression. It is also unclear whether continued exposure to the individualized FPA during daily walking is necessary to maintain reductions in knee adduction moment, or whether participants would revert to their pre-training movement patterns once supervised feedback is removed. Prior gait retraining and neuromuscular exercise studies suggest that long-term retention of biomechanical changes may require either extended training durations, periodic booster sessions, or integration of the learned movement strategies into everyday functional activities. Future studies should therefore examine longer-term follow-up periods (e.g., 3–12 months) and investigate maintenance strategies such as home-based training, to promote sustained adoption of beneficial gait and stability patterns.

## V. Conclusion

In conclusion, this study demonstrates that a six-week off-axis elliptical training program with individualized FPA feedback can significantly reduce the knee adduction moment, improve frontal-plane stability, and enhance certain clinical and functional outcomes in individuals with medial KOA. This personalized, biomechanically guided exercise approach appears to be a promising non-invasive option for managing medial compartment KOA. Our findings add to the growing body of evidence supporting targeted exercise interventions for knee OA and provide a foundation for future research on long-term effects and underlying mechanisms of such training. Incorporating off-axis elliptical stepping training into rehabilitation programs may offer a safe and functional method to reduce painful knee loading, improve stability and function, and potentially slow disease progression—ultimately improving quality of life for individuals with medial knee osteoarthritis.

## Figures and Tables

**TABLE I:** KOOS at Baseline, Post-Training, and Follow-Up

Measures	Baseline	Post-Training	Follow up
KOOS Symptoms	63.89 (15.09)	74.21 (13.10) *	70.54 (19.54)
KOOS Pain	66.98 (18.45)	75.62 (18.26)	70.83 (22.17)
KOOS ADL	75.00 (19.43)	83.33 (19.12)	75.37 (28.91)
KOOS Sport	45.00 (37.08)	45.00 (30.41)	46.25 (36.82)
KOOS QOL	40.28 (29.50)	43.75 (23.59)	43.75 (28.74)

* Significantly different from baseline, p-value < 0.05.

**TABLE II:** Functional Outcome Measures (Mean (SD))

Measures	Baseline	Post-Training	Follow up
TUG (s)	11.12 (1.27)	9.82 (1.60)	10.51 (1.55)
20MWT(s)	19.36(2.24)	17.33(3.15)	16.94 (4.84)
STS (count)	8.13 (2.99)	9.50 (3.16)*	9.67 (2.81)
FSST (s)	9.50 (1.12)	8.50 (0.88)	8.67 (0.94)

TUG: Timed up and go test, 20MWT: 20-meter walk test, STS = 30-second sit to stand test, FSST = four square step tests.

* Significantly different from baseline, p-value < 0.05.

**TABLE III:** Spatiotemporal Gait Characteristics During the Different Evaluations

Measures	Baseline	Post-Training	Follow up
Velocity (m/s)	1.09(0.23)	1.18(0.25) *	1.10(0.23)
*Step Length (cm)*			
Right lower limb	62.40(9.65)	64.97(9.27)	66.03(7.98)
Left lower limb	62.84(9.38)	65.52(8.42)	65.80(7.99)
*Single support phase (%)*			
Right lower limb	34.34(2.47)	35.27(1.36)	35.60(1.18)
Left lower limb	34.54(2.60)	35.22(1.94)	35.90(1.58)
Single support phase symmetry	0.99(0.05)	1.00 (0.04)	0.99(0.03)

* Significantly different from baseline, p-value < 0.05.

## References

[1] Wise BL , “Functional impairment is a risk factor for knee replacement in the multicenter osteoarthritis study,” Clin. Orthopaedics Rel. Res, vol. 473, no. 8, pp. 2505–2513, 2015, doi: 10.1007/s11999-015-4211-3. PMC4488226 25754756

[2] Mündermann A, Dyrby CO, Hurwitz DE, Sharma L, and Andriacchi TP, “Potential strategies to reduce medial compartment loading in patients with knee osteoarthritis of varying severity: Reduced walking speed,” Arthritis Rheumatism, vol. 50, no. 4, pp. 1172–1178, Apr. 2004, doi: 10.1002/art.20132. 15077299

[3] Sharma L , “Knee adduction moment, serum hyaluronan level, and disease severity in medial tibiofemoral osteoarthritis,” Arthritis Rheumatism, vol. 41, no. 7, pp. 1233–1240, Jul. 1998, doi: 10.1002/1529-0131(199807)41:7<1233:aid-art14>3.0.co;2-l. 9663481

[4] Hunt MA and Takacs J, “Effects of a 10-week toe-out gait modification intervention in people with medial knee osteoarthritis: A pilot, feasibility study,” Osteoarthritis Cartilage, vol. 22, no. 7, pp. 904–911, Jul. 2014, doi: 10.1016/j.joca.2014.04.007. 24836210

[5] Hunt MA, Charlton JM, Krowchuk NM, Tse CTF, and Hatfield GL, “Clinical and biomechanical changes following a 4-month toe-out gait modification program for people with medial knee osteoarthritis: A randomized controlled trial,” Osteoarthritis Cartilage, vol. 26, no. 7, pp. 903–911, Jul. 2018, doi: 10.1016/j.joca.2018.04.010. 29709498

[6] Hunt MA, Simic M, Hinman RS, Bennell KL, and Wrigley TV, “Feasibility of a gait retraining strategy for reducing knee joint loading: Increased trunk lean guided by real-time biofeedback,” J. Biomechanics, vol. 44, no. 5, pp. 943–947, Mar. 2011, doi: 10.1016/j.jbiomech.2010.11.027. 21144522

[7] Shull PB , “Toe-in gait reduces the first peak knee adduction moment in patients with medial compartment knee osteoarthritis,” J. Biomechanics, vol. 46, no. 1, pp. 122–128, Jan. 2013, doi: 10.1016/j.jbiomech.2012.10.019. 23146322

[8] Shull PB, Lurie KL, Cutkosky MR, and Besier TF, “Training multi-parameter gaits to reduce the knee adduction moment with data-driven models and haptic feedback,” J. Biomechanics, vol. 44, no. 8, pp. 1605–1609, May 2011, doi: 10.1016/j.jbiomech.2011.03.016. 21459384

[9] Uhlrich SD, Silder A, Beaupre GS, Shull PB, and Delp SL, “Subject-specific toe-in or toe-out gait modifications reduce the larger knee adduction moment peak more than a non-personalized approach,” J. Biomechanics, vol. 66, pp. 103–110, Jan. 2018, doi: 10.1016/j.jbiomech.2017.11.003. PMC5859947 29174534

[10] Uhlrich SD , “Personalization improves the biomechanical efficacy of foot progression angle modifications in individuals with medial knee osteoarthritis,” J. Biomechanics, vol. 144, Nov. 2022, Art. no. 111312, doi: 10.1016/j.jbiomech.2022.111312. PMC9889103 36191434

[11] Simic M, Wrigley TV, Hinman RS, Hunt MA, and Bennell KL, “Altering foot progression angle in people with medial knee osteoarthritis: The effects of varying toe-in and toe-out angles are mediated by pain and malalignment,” Osteoarthritis Cartilage, vol. 21, no. 9, pp. 1272–1280, Sep. 2013. 23973141 10.1016/j.joca.2013.06.001

[12] Baghi R , “Determining individualized foot progression angle for reduction of knee medial compartment loading during stepping,” Med. Sci. Sports Exercise, vol. 57, no. 1, pp. 33–43, Jan. 2025, doi: 10.1249/mss.0000000000003531. PMC12411098 39186734

[13] Sharma L, Cahue S, Song J, Hayes K, Pai Y-C, and Dunlop D, “Physical functioning over three years in knee osteoarthritis: Role of psychosocial, local mechanical, and neuromuscular factors,” Arthritis Rheumatism, vol. 48, no. 12, pp. 3359–3370, Dec. 2003, doi: 10.1002/art.11420. 14673987

[14] Lewek MD, Ramsey DK, Snyder-Mackler L, and Rudolph KS, “Knee stabilization in patients with medial compartment knee osteoarthritis,” Arthritis Rheumatism, vol. 52, no. 9, pp. 2845–2853, Sep. 2005, doi: 10.1002/art.21237. 16142714 PMC1343471

[15] Bennell KL, Bowles K-A, Wang Y, Cicuttini F, Davies-Tuck M, and Hinman RS, “Higher dynamic medial knee load predicts greater cartilage loss over 12 months in medial knee osteoarthritis,” Ann. Rheumatic Diseases, vol. 70, no. 10, pp. 1770–1774, Oct. 2011, doi: 10.1136/ard.2010.147082. 21742637

[16] Rutherford D, Baker M, Urquhart N, and Stanish W, “The effect of a frontal plane gait perturbation bout on knee biomechanics and muscle activation in older adults and individuals with knee osteoarthritis,” Clin. Biomechanics, vol. 92, Feb. 2022, Art. no. 105574, doi: 10.1016/j.clinbiomech.2022.105574. 35066441

[17] Fregly BJ, Reinbolt JA, Rooney KL, Mitchell KH, and Chmielewski TL, “Design of patient-specific gait modifications for knee osteoarthritis rehabilitation,” IEEE Trans. Biomed. Eng, vol. 54, no. 9, pp. 1687–1695, Sep. 2007. 17867361 10.1109/TBME.2007.891934 PMC2040055

[18] Mündermann A, Asay JL, Mündermann L, and Andriacchi TP, “Implications of increased medio-lateral trunk sway for ambulatory mechanics,” J. Biomechanics, vol. 41, no. 1, pp. 165–170, 2008. 10.1016/j.jbiomech.2007.07.001 17678933

[19] Willson JD, Ratcliff OM, Meardon SA, and Willy RW, “Influence of step length and landing pattern on patellofemoral joint kinetics during running,” Scandin. J. Med. Sci. Sports, vol. 25, no. 6, pp. 736–743, Dec. 2015. 10.1111/sms.12383 25585589

[20] Liao T-C, Yang N, Ho K-Y, Farrokhi S, and Powers CM, “Femur rotation increases patella cartilage stress in females with patellofemoral pain,” Med. Sci. Sports Exercise, vol. 47, no. 9, pp. 1775–1780, 2015. 10.1249/MSS.0000000000000617 25606814

[21] Andriacchi TP, Mündermann A, Smith RL, Alexander EJ, Dyrby CO, and Koo S, “A framework for the in vivo pathomechanics of osteoarthritis at the knee,” Ann. Biomed. Eng, vol. 32, no. 3, pp. 447–457, Mar. 2004. 15095819 10.1023/b:abme.0000017541.82498.37

[22] Barrios JA, Crossley KM, and Davis IS, “Gait retraining to reduce the knee adduction moment through real-time visual feedback of dynamic knee alignment,” J. Biomechanics, vol. 43, no. 11, pp. 2208–2213, Aug. 2010, doi: 10.1016/j.jbiomech.2010.03.040. PMC2914211 20452595

[23] Manal K, Gardinier E, Buchanan TS, and Snyder-Mackler L, “A more informed evaluation of medial compartment loading: The combined use of the knee adduction and flexor moments,” Osteoarthritis Cartilage, vol. 23, no. 7, pp. 1107–1111, Jul. 2015, doi: 10.1016/j.joca.2015.02.779. 25862486 PMC4470852

[24] Richards RE, Andersen MS, Harlaar J, and van den Noort JC, “Relationship between knee joint contact forces and external knee joint moments in patients with medial knee osteoarthritis: Effects of gait modifications,” Osteoarthritis Cartilage, vol. 26, no. 9, pp. 1203–1214, Sep. 2018. 29715509 10.1016/j.joca.2018.04.011

[25] Kang SH, Lee SJ, Press JM, and Zhang L-Q, “Real-time three-dimensional knee moment estimation in knee osteoarthritis: Toward biodynamic knee osteoarthritis evaluation and training,” IEEE Trans. Neural Syst. Rehabil. Eng, vol. 27, no. 6, pp. 1263–1272, Jun. 2019, doi: 10.1109/TNSRE.2019.2915812. 31071049 PMC6697482

[26] Kang SH, Lee SJ, and Zhang L-Q, “Real-time tracking of knee adduction moment in patients with knee osteoarthritis,” J. Neurosci. Methods, vol. 231, pp. 9–17, Jul. 2014, doi: 10.1016/j.jneumeth.2013.12.001. 24361759 PMC4061264

[27] Ren Y, Lee SJ, Park H-S, and Zhang L-Q, “A pivoting elliptical training system for improving pivoting neuromuscular control and rehabilitating musculoskeletal injuries,” IEEE Trans. Neural Syst. Rehabil. Eng, vol. 21, no. 5, pp. 860–868, Sep. 2013, doi: 10.1109/TNSRE.2013.2273874. 24013591

[28] Lee SJ, Ren Y, Press JM, Lee J, and Zhang L-Q, “Improvement in offaxis neuromuscular control under slippery conditions following six-week pivoting leg neuromuscular training,” IEEE Trans. Neural Syst. Rehabil. Eng, vol. 25, no. 11, pp. 2084–2093, Nov. 2017, doi: 10.1109/TNSRE.2017.2705664. 28541212

[29] Kang SH, Lee SJ, Ren Y, and Zhang L-Q, “Real-time knee adduction moment feedback training using an elliptical trainer,” IEEE Trans. Neural Syst. Rehabil. Eng, vol. 22, no. 2, pp. 334–343, Mar. 2014, doi: 10.1109/TNSRE.2013.2291203. 24608687

[30] Miyazaki T, Wada M, Kawahara H, Sato M, Baba H, and Shimada S, “Dynamic load at baseline can predict radiographic disease progression in medial compartment knee osteoarthritis,” Ann. Rheumatic Diseases, vol. 61, no. 7, pp. 617–622, Jul. 2002, doi: 10.1136/ard.61.7.617. PMC1754164 12079903

[31] Chehab EF, Favre J, Erhart-Hledik JC, and Andriacchi TP, “Baseline knee adduction and flexion moments during walking are both associated with 5 year cartilage changes in patients with medial knee osteoarthritis,” Osteoarthritis Cartilage, vol. 22, no. 11, pp. 1833–1839, Nov. 2014, doi: 10.1016/j.joca.2014.08.009. 25211281 PMC4369510

[32] Lu T-W, Chien H-L, and Chenü H-L, “Joint loading in the lower extremities during elliptical exercise,” Med. Sci. Sports Exercise, vol. 39, no. 9, pp. 1651–1658, Sep. 2007, doi: 10.1249/mss.0b013e3180dc9970. 17805099

[33] Burnfield JM, Shu Y, Buster T, and Taylor A, “Similarity of joint kinematics and muscle demands between elliptical training and walking: Implications for practice,” Phys. Therapy, vol. 90, no. 2, pp. 289–305, Feb. 2010, doi: 10.2522/ptj.20090033. 20022994

[34] Lee SJ, Ren Y, Geiger F, and Zhang L-Q, “Gender differences in offaxis neuromuscular control during stepping under a slippery condition,” Eur. J. Appl. Physiol, vol. 113, no. 11, pp. 2857–2866, Nov. 2013, doi: 10.1007/s00421-013-2727-3. 24062010 PMC3828294

[35] Lee SJ, Ren Y, Chang AH, Press JM, Hochberg MC, and Zhang L-Q, “Plane dependent subject-specific neuromuscular training for knee rehabilitation,” IEEE Trans. Neural Syst. Rehabil. Eng, vol. 28, no. 8, pp. 1876–1883, Aug. 2020, doi: 10.1109/TNSRE.2020.3005119. 32746305 PMC7489920

[36] Lee SJ, Jin D, Kang SH, Gaebler-Spira D, and Zhang L-Q, “Combined Ankle/Knee stretching and pivoting stepping training for children with cerebral palsy,” IEEE Trans. Neural Syst. Rehabil. Eng, vol. 27, no. 9, pp. 1743–1752, Sep. 2019, doi: 10.1109/TNSRE.2019.2934139. 31403432 PMC6779478

[37] Lee SJ, Ren Y, Chang AH, Geiger F, and Zhang L-Q, “Effects of pivoting neuromuscular training on pivoting control and proprioception,” Med. Sci. Sports Exercise, vol. 46, no. 7, pp. 1400–1409, 2014, doi: 10.1249/mss.0000000000000249. PMC4140528 24389517

[38] Tsai L-C, Ren Y, Gaebler-Spira DJ, Revivo GA, and Zhang L-Q, “Effects of an off-axis pivoting elliptical training program on gait function in persons with spastic cerebral palsy: A preliminary study,” Amer. J. Phys. Med. Rehabil, vol. 96, no. 7, pp. 515–522, 2017, doi: 10.1097/phm.0000000000000632. 28628539

[39] Tsai L-C, Lee SJ, Yang AJ, Ren Y, Press JM, and Zhang L-Q, “Effects of off-axis elliptical training on reducing pain and improving knee function in individuals with patellofemoral pain,” Clin. J. Sport Med, vol. 25, no. 6, pp. 487–493, 2015, doi: 10.1097/jsm.0000000000000164. 25591131 PMC4501904

[40] Zhang W , “OARSI recommendations for the management of hip and knee osteoarthritis,” Osteoarthritis Cartilage, vol. 18, no. 4, pp. 476–499, Apr. 2010, doi: 10.1016/j.joca.2010.01.013. 20170770

[41] Fransen M, McConnell S, Harmer AR, Van der Esch M, Simic M, and Bennell KL, “Exercise for osteoarthritis of the knee: A cochrane systematic review,” Brit. J. Sports Med, vol. 49, no. 24, pp. 1554–1557, Dec. 2015, doi: 10.1136/bjsports-2015-095424. 26405113

[42] Bao X, Tan J, Flyzik M, Ma X, Liu H, and Liu H, “Effect of therapeutic exercise on knee osteoarthritis after intra-articular injection of botulinum toxin type A, hyaluronate or saline: A randomized controlled trial,” J. Rehabil. Med, vol. 50, no. 6, pp. 534–541, 2018, doi: 10.2340/16501977-2340. 29664106

[43] Roos EM and Lohmander LS, “The Knee injury and Osteoarthritis Outcome Score (KOOS): From joint injury to osteoarthritis,” Health Quality Life Outcomes, vol. 1, pp. 1–8, Nov. 2003. 10.1186/1477-7525-1-64 PMC280702 14613558

[44] Engelhart L , “Validation of the knee injury and osteoarthritis outcome score subscales for patients with articular cartilage lesions of the knee,” Amer. J. Sports Med, vol. 40, no. 10, pp. 2264–2272, Oct. 2012, doi: 10.1177/0363546512457646. 22962288

[45] Debi R , “Differences in gait patterns, pain, function and quality of life between males and females with knee osteoarthritis: A clinical trial,” BMC Musculoskeletal Disorders, vol. 10, no. 1, pp. 1–10, Dec. 2009, doi: 10.1186/1471-2474-10-127. 19825163 PMC2765955

[46] Al-Khlaifat L, Herrington LC, Hammond A, Tyson SF, and Jones RK, “The effectiveness of an exercise programme on knee loading, muscle co-contraction, and pain in patients with medial knee osteoarthritis: A pilot study,” Knee, vol. 23, no. 1, pp. 63–69, Jan. 2016, doi: 10.1016/j.knee.2015.03.014. 25953672

